# Accelerating precision biology and medicine with computational biology and bioinformatics

**DOI:** 10.1186/s13059-014-0450-y

**Published:** 2014-09-05

**Authors:** Yves A Lussier, Haiquan Li, Nima Pouladi, Qike Li

**Affiliations:** Department of Medicine, University of Arizona, 1501 N Campbell Avenue, Tucson, AZ 85724 USA; BIO5 Institute, University of Arizona, 1657 E Helen Street, Tucson, AZ 85721 USA; University of Arizona Cancer Center, 1515 N Campbell Avenue, Tucson, AZ 85724 USA; Interdisciplinary Program in Statistics, University of Arizona, 617 North Santa Rita Avenue, Tucson, AZ 85721 USA

## Abstract

A report on the 22^nd^ Annual International Conference on Intelligent Systems for Molecular Biology, held in Boston, Massachusetts, USA, July 11-15, 2014.

## Meeting report

For the first time this year at the Intelligent Systems for Molecular Biology conference, special talks honored and celebrated the Nobel laureates of Chemistry 2013 (computational and theoretical) Arieh Warshel, Martin Karplus and Michael Levitt. In addition, keynote speakers included Michal Linial, Eugene (Gene) Myers, Isaac (Zak) Kohane, Dana Pe’er, Robert Langer, and Russ B Altman. The conference provided insight into innovative computational methods, such as single-cell sequencing analyses that enable unprecedented resolution for the study of disease heterogeneity, rare cell aberrations and the microbiome. Furthermore, biomedical studies were enhanced with more data-driven projects across multiple scales, including Encyclopedia of DNA Elements (ENCODE) and The Cancer Genome Atlas; therefore, it seems that data integration remains the bottleneck of studies rather than data generation.

## Next- and third-generation sequencing and single-cell sequencing

The industry has witnessed the progression of sequencing to next-generation techniques and then a third generation, which can potentially enhance the measurement of single-cell genomes. However, single-cell sequencing might induce analytical challenges as it leads to overlapping paths in the *de novo* assembly graphs. The algorithm ExSPAnder, reported by Andrey Prjibelski (St Petersburg Academic University, Russia), addresses this issue by mapping each path, which consists of paired-end fragments including repetitive ones, into a two-dimensional rectangular space and by modeling the correct path as a dense parallel region in the rectangle. Mikael Boden (The University of Queensland, Australia) presented a Bayesian inference method, STRViper, to estimate repeat-length variation from the paired-end short reads. This method incorporates prior knowledge of variation in short tandem repeats (STRs). The longer-range positional information provided by paired-end reads facilitates the identification of STRs sized beyond the standard read length. Adam Phillippy (National Biodefense Analysis and Countermeasures Center, USA) highlighted a PacBio-based method that improves the accuracy of third-generation sequencing by combining the long reads with high-fidelity short reads yielded from next-generation sequencing techniques. This corrects random errors in the long reads by using the information of the short reads and improves the subsequent assembly of corrected long reads.

Classic *de novo* sequence assembly usually works for reads from a single genome; nonetheless, studies show that combinations of multiple genomes could improve accuracies of target genome assembly. Mikhail Kolmogorov (Saint-Petersburg Academic University, Russia) presented ‘Ragout’ that works on multiple reference genomes to help determine the order of contigs. Specifically, Kolmogorov employed the evolutionarily conserved synteny blocks on these reference genomes and constructed a breakpoint graph by which a parsimony model was employed to estimate the order of contigs.

In RNA-Seq techniques, Zhaojun Zhang (University of Carolina at Chapel Hill, USA) indicated that the computationally expensive fragment-alignment step could be bypassed. In his algorithm named ‘RNA-Skim’, the abundance of transcripts can be estimated by means of a new concept – ‘sig-mers’ – which is defined as unique k-mers of transcript clusters. Zhang demonstrated that scanning sig-mers could derive approximate transcript levels with exceptional speed. With more RNA-Seq analysis in practice, benchmarking is crucial. David Kreil (Boku University Vienna, Austria) discussed the latest advance of the sequencing quality control consortium (SEQC/MAQC-III) on performance study of RNA-seq techniques by using different algorithms.

## Translational bioinformatics

Drug repositioning – identifying new targets and diseases for existing compounds – remains a clinically valuable endeavor for translational bioinformatics. James Costello (University of Colorado, USA) presented a community effort that assessed 44 algorithms predicting drug response in human breast cancer cell lines using a combination of genomic, epigenomic and proteomic profiles. Modeling the nonlinearities in the data and incorporating prior knowledge, such as biological pathways, improved the overall performance. Additionally, gene expression data carry the most informative signal, and methylation is the most non-redundant signal.

Employment of single-targeted therapies has led to promising results in cancer treatment, even though evolving resistance hinders their clinical efficacy. However, targeting several pathways with multiple therapeutic agents addresses this challenge. Martin Miller (Memorial Sloan Kettering Cancer Center, USA) gave a presentation on how his group had identified the cyclin-dependent kinase CDK4 and the insulin-like growth factor receptor IGF1R as synergistic drug targets for treating de-differentiated liposarcoma. Their approach integrates combinatorial drug perturbation of tumor cells and quantifying the viability of tumor cells with network modeling. Importantly, they showed that their quantitative approach is capable of accurately predicting the degree of viability of tumor cells after pairwise-perturbing the nodes in their network models. They also validated their quantitative model *in vitro*. Lei Huang (Peking University, China) developed a computational tool – DrugComboRanker – to prioritize synergistic drug combinations by finding co-targeted alternative and complementary signaling modules. These modules are constructed using patients’ gene expression profiles and interactome data in the context of a disease.

Invasive prenatal procedures harbor a 1% risk of miscarriage. WISECONDOR, developed by Roy Straver and colleagues (VU University, Netherlands), leverages low-coverage next-generation sequencing to detect chromosomal aberrations of the fetus noninvasively. Applying within-sample comparison schemes, they compared GC-normalized reads of the target region with a group of reference regions on other chromosomes that behave similarly. The bins that deviate significantly from the reference regions are considered as being potentially aberrant.

Effective precision medicine requires an improved understanding of tumor heterogeneity. Mark Leiserson (Brown University, USA) developed a computational algorithm called multi-dendrix by which he analyzed the mutation data from glioblastoma, breast and lung adenocarcinoma cancers. Multi-dendrix incorporates the concept of driver pathways and simultaneously identifies multiple driver pathways with different sizes without any prior knowledge of currently known pathways. Giovanni Ciriello (Memorial Sloan-Kettering Cancer Center, USA) segregated 3,299 samples of 12 distinct tumors apart from their tissue of origin into two distinct genomic classes using bipartite graphs and module analyses. These classes – (i) *TP53* mutations and copy-number variants versus (ii) somatic mutations – also segregate into more subclasses. By contrast, Matan Hofree (University of California, San Diego, USA) and colleagues developed a network-based classification strategy for ovarian, uterine and lung cancers. They mapped intragenic mutations onto the protein-protein interaction networks. Patients were further classified into various subtypes by using a non-negative matrix-factorization algorithm, corroborated by simulation and cross validation. Teresa Przytycka (National Institutes of Health, USA) and colleagues also approached this challenge by developing a novel probabilistic method and integrating genetic aberrations and expression values of genes. Their method also identifies patients with similar phenotypic characteristics who have similar underlying genetic aberrations. Importantly, the algorithm does not assign a unique discrete subtype to each individual patient but establishes a probability between each patient and each subtype.

The advent of high-throughput sequencing technologies enables an understanding of the genomic architecture of heterogeneous populations of cells, thus estimating the frequency of various subclones of tumor cells. Iman Hajirasouliha (Brown University, USA) formulated the clonal expansions of tumor cells with a rooted binary tree in which the nodes correspond to different populations of tumor cells with a distinctive mutation profile. The edges also represent their ancestral relationships. Their binary tree partition algorithm reduces the intrinsic computational challenge. Quaid Morris (University of Toronto, Canada) presented PhyloSub for modeling the clonal evolutionary pattern of tumors. PhyloSub uses a nonparametric Bayesian inference based on Markov chain Monte Carlo sampling and employs the Dirichlet process as the prior distribution over trees conducted over the single-nucleotide variant (SNV) frequency data of one or multiple tumor samples. He also presented an additional model to address the challenge of multiple phylogenies derived from the SNV frequency data.

## Metagenomics and metaproteomics of microbiomes

The human microbiome is becoming increasingly relevant to the understanding of the pathophysiology of diseases. Of note, Eric Franzosa (Harvard School of Public Health, USA) collected stool and saliva samples from healthy individuals and uncovered a discordancy between their metagenomic DNA count and metatranscriptomic mRNA expression profile. Furthermore, he found a small number of oral species that are genomically present and yet transcriptionally inactive in the gut microbiota and vice versa. Kristoffer Forslund (European Molecular Biology Laboratory, Germany) developed a population-scale metric to assess, in gut metagenomes, the antibiotic resistance potential associated with consumption by food type and country. He found a positive correlation between antibiotic use in animals and evolving resistance of human gut microbiota that is country specific. By contrast, metaproteomics has a role in disease diagnoses but is limited by the presence of highly similar protein sequences among related organisms. Bernhard Renard (Robert Koch Institute, Germany) presented an algorithm named Pipasic that corrects peptide proteome abundance after modeling the aggregated abundance by similar protein sequences. He demonstrated more-accurate quantification of peptides for mixed species in comparison with alternative methods.

## ENCODE-driven analytics and data integration

Since its release in 2012, the innovative data-driven project known as ENCODE has led to many ongoing studies. As reflected in more than 20 posters and talks at the conference, this abundant data source can be re-used to model biological systems, to generate hypotheses or discoveries in high throughput, and to confirm pre-existing hypotheses. For instance, Andrew Johnson (National Institute of Health, USA) built a comprehensive database, named GRASP, that was derived from 1,390 genome-wide association studies (GWASs) and included more genotype-phenotype associations than the National Human Genome Research Institute GWAS catalogue, including expression quantitative trait loci (eQTLs), methylation QTLs and metabolite QTLs. Johnson demonstrated an example that combined histone and ChiP-Seq signatures in ENCODE to validate the function of trait-associated single-nucleotide polymorphisms. Additionally, Yuanfang Guan (University of Michigan, USA) employed the RNA-Seq data of ENCODE to predict the distinct functions of each isoform of a gene. Using a multiple-instance learning technique in conjunction with a support vector machine, they consider a gene as a ‘bag’ of multiple isoform instances with potentially different functions. Thereafter, they can learn the common-feature model of a subset of the isoforms across all genes involved in the specific gene ontology term function under consideration.

Finally, two studies used genome-wide chromosome conformation capture data that provide information on long-range interactions among chromatins. Using maximum-likelihood approaches, Noam Kaplan (University of Massachusetts, USA) predicted the locus of 65 human genome contigs, for which the alignment was unknown. Jianlin Cheng (University of Missouri Columbia, USA) predicted the three-dimensional structure of whole human chromosomes and unveiled important structural conformations that could be related to mechanisms underlying malignant B-cell acute lymphoblastic leukemias.

## Abbreviations

ENCODE: Encyclopedia of DNA Elements
eQTL: expression quantitative trait locus
GWAS: Genome-wide association study
QTL: Quantitative trait locus
SNV: Single-nucleotide variant
STR: Short tandem repeat

